# *N*-(3,4-Dichloro­phen­yl)-4-methyl­benzene­sulfonamide

**DOI:** 10.1107/S1600536810051305

**Published:** 2010-12-11

**Authors:** K. Shakuntala, Sabine Foro, B. Thimme Gowda

**Affiliations:** a Department of Chemistry, Mangalore University, Mangalagangotri 574 199, Mangalore, India; b Institute of Materials Science, Darmstadt University of Technology, Petersenstrasse 23, D-64287 Darmstadt, Germany

## Abstract

In the title compound, C_13_H_11_Cl_2_NO_2_S, the conformation of the N—C bond in the C—SO_2_—NH—C segment has *gauche* torsions with respect to the S=O bonds. The mol­ecule is bent at the S atom with a C—SO_2_—NH—C torsion angle of 64.3 (4)°. Furthermore, the conformation of the N—H bond and the *meta*-chloro group in the adjacent benzene ring are *anti* to each other. The two benzene rings are tilted relative to each other by 82.5 (1)°. In the crystal, mol­ecules are linked by pairs of N—H⋯O(S) hydrogen bonds, forming inversion dimers.

## Related literature

For our study of the effect of substituents on the structures of *N*-(ar­yl)aryl­sulfonamides, see: Gowda *et al.* (2009 ▶); Shakuntala *et al.* (2010 ▶, 2011 ▶); For related structures, see: Gelbrich *et al.* (2007 ▶); Perlovich *et al.* (2006 ▶).
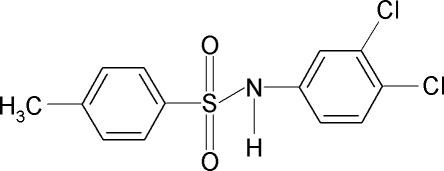


## Experimental

### 

#### Crystal data

C_13_H_11_Cl_2_NO_2_S
*M* *_r_* = 316.19
Monoclinic, 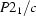

*a* = 9.543 (1) Å
*b* = 13.628 (2) Å
*c* = 10.893 (1) Å
β = 94.85 (1)°
*V* = 1411.6 (3) Å^3^
*Z* = 4
Cu *K*α radiation
μ = 5.50 mm^−1^
*T* = 299 K
0.40 × 0.28 × 0.18 mm

#### Data collection

Enraf–Nonius CAD-4 diffractometer
Absorption correction: ψ scan (North *et al.*, 1968 ▶) *T*_min_ = 0.217, *T*_max_ = 0.438
2927 measured reflections
2462 independent reflections
1791 reflections with *I* > 2σ(*I*)
*R*_int_ = 0.117
3 standard reflections every 120 min  intensity decay: 1.0%

#### Refinement

*R*[*F* ^2^ > 2σ(*F* ^2^)] = 0.086
*wR*(*F* ^2^) = 0.236
*S* = 1.03
2462 reflections
176 parameters
1 restraint
H atoms treated by a mixture of independent and constrained refinement
Δρ_max_ = 0.38 e Å^−3^
Δρ_min_ = −0.97 e Å^−3^

### 

Data collection: *CAD-4-PC* (Enraf–Nonius, 1996 ▶); cell refinement: *CAD-4-PC*; data reduction: *REDU4* (Stoe & Cie, 1987 ▶); program(s) used to solve structure: *SHELXS97* (Sheldrick, 2008 ▶); program(s) used to refine structure: *SHELXL97* (Sheldrick, 2008 ▶); molecular graphics: *PLATON* (Spek, 2009 ▶); software used to prepare material for publication: *SHELXL97*.

## Supplementary Material

Crystal structure: contains datablocks I, global. DOI: 10.1107/S1600536810051305/ds2077sup1.cif

Structure factors: contains datablocks I. DOI: 10.1107/S1600536810051305/ds2077Isup2.hkl

Additional supplementary materials:  crystallographic information; 3D view; checkCIF report

## Figures and Tables

**Table 1:** Hydrogen-bond geometry (Å, °)

*D*—H⋯*A*	*D*—H	H⋯*A*	*D*⋯*A*	*D*—H⋯*A*
N1—H1*N*⋯O2^i^	0.83 (3)	2.10 (3)	2.908 (5)	166 (5)

Symmetry code: (i) 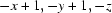.

**Fig. 1.**
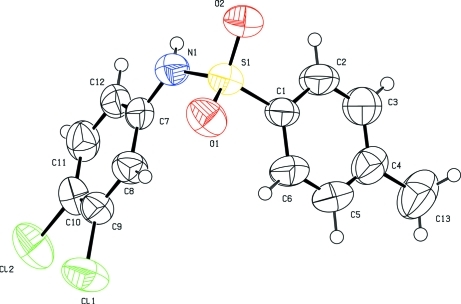
Molecular structure of (I), showing the atom labelling scheme and displacement ellipsoids are drawn at the 50% probability level.

**Fig. 2.**
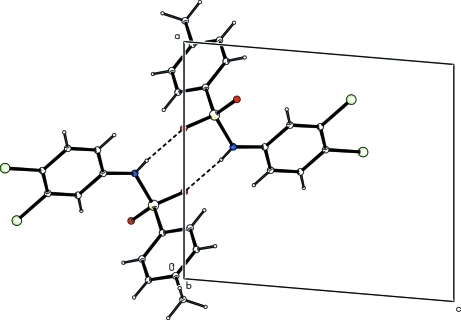
Molecular packing of (I) with hydrogen bonding shown as dashed lines.

## supplementary crystallographic information

### Comment

As part of a study of the substituent effects on the crystal structures of *N*-(aryl)-arylsulfonamides (Gowda *et al.*, 2009; Shakuntala *et al.*, 2010; 2011), in the present work, the structure of *N*-(3,4-dichlorophenyl)-4-methylbenzenesulfonamide (I) has been determined. In (I), the conformation of the N—C bond in the C—SO_2_—NH—C segment has *gauche* torsions with respect to the S═O bonds (Fig. 1). The conformation of the N—H bond and the *meta*-chloro groups in the adjacent benzene ring are *anti* to each other.

The molecule is bent at the S atom with the C—SO_2_—NH—C torsion angle of 64.3 (4)°, compared to the values of 65.4 (2)° (molecule 1) and -61.7 (2)° (molecule 2) in *N*-(2,3-dichlorophenyl)-4- methylbenzenesulfonamide (II) (Shakuntala *et al.*, 2010), 62.1 (2)° in *N*-(2,5-dichlorophenyl)-4-methylbenzenesulfonamide (III) (Shakuntala *et al.*, 2011) and 69.3 (4)° in *N*-(3,5-dichlorophenyl)-4-methylbenzenesulfonamide (IV) (Gowda *et al.*, 2009).

The benzene rings in the title compound are tilted relative to each other by 82.5 (1)°, compared to the values of 76.0 (1)° (molecule 1) and 79.9 (1)° (molecule 2) in (II), 67.8 (1)° in (III) and 79.6 (1)° in (IV).

The other bond parameters in (I) are similar to those observed in (II), (III), (IV) and other aryl sulfonamides (Perlovich *et al.*, 2006; Gelbrich *et al.*, 2007).

The packing of molecules linked by of N—H···O(S) hydrogen bonds(Table 1) is shown in Fig. 2.

### Experimental

The solution of toluene (10 ml) in chloroform (40 ml) was treated dropwise with chlorosulfonic acid (25 ml) at 0 ° C. After the initial evolution of hydrogen chloride subsided, the reaction mixture was brought to room temperature and poured into crushed ice in a beaker. The chloroform layer was separated, washed with cold water and allowed to evaporate slowly. The residual 4-methylbenzenesulfonylchloride was treated with 3,4-dichloroaniline in the stoichiometric ratio and boiled for ten minutes. The reaction mixture was then cooled to room temperature and added to ice cold water (100 ml). The resultant *N*-(3,4-dichlorophenyl)-4-methylbenzenesulfonamide was filtered under suction and washed thoroughly with cold water. It was then recrystallized to constant melting point from dilute ethanol. The purity of the compound was checked and characterized by recording its infrared and NMR spectra.

Prism like light brown single crystals used in X-ray diffraction studies were grown in ethanolic solution by slow evaporation at room temperature.

### Refinement

The H atom of the NH group was located in a difference map and later restrained to the distance N—H = 0.86 (3) Å. The other H atoms were positioned with idealized geometry using a riding model with C—H = 0.93–0.96 Å A l l H atoms were refined with isotropic displacement parameters (set to 1.2 times of the *U*_eq_ of the parent atom).

### Crystal data

**Table:**

C_13_H_11_Cl_2_NO_2_S	*F*(000) = 648
*M**_r_* = 316.19	*D*_x_ = 1.488 Mg m^−^^3^
Monoclinic, *P*2_1_/*c*	Cu *K*α radiation, λ = 1.54180 Å
Hall symbol: -P 2ybc	Cell parameters from 25 reflections
*a* = 9.543 (1) Å	θ = 5.7–18.6°
*b* = 13.628 (2) Å	µ = 5.50 mm^−^^1^
*c* = 10.893 (1) Å	*T* = 299 K
β = 94.85 (1)°	Prism, light brown
*V* = 1411.6 (3) Å^3^	0.40 × 0.28 × 0.18 mm
*Z* = 4	

### Data collection

**Table:**

Enraf–Nonius CAD-4 diffractometer	1791 reflections with *I* > 2σ(*I*)
Radiation source: fine-focus sealed tube	*R*_int_ = 0.117
graphite	θ_max_ = 66.9°, θ_min_ = 4.7°
ω/2θ scans	*h* = −11→2
Absorption correction: ψ scan (North *et al.*, 1968)	*k* = −16→0
*T*_min_ = 0.217, *T*_max_ = 0.438	*l* = −13→13
2927 measured reflections	3 standard reflections every 120 min
2462 independent reflections	intensity decay: 1.0%

### Refinement

**Table:**

Refinement on *F*^2^	Primary atom site location: structure-invariant direct methods
Least-squares matrix: full	Secondary atom site location: difference Fourier map
*R*[*F*^2^ > 2σ(*F*^2^)] = 0.086	Hydrogen site location: inferred from neighbouring sites
*wR*(*F*^2^) = 0.236	H atoms treated by a mixture of independent and constrained refinement
*S* = 1.03	*w* = 1/[σ^2^(*F*_o_^2^) + (0.1697*P*)^2^ + 0.0915*P*] where *P* = (*F*_o_^2^ + 2*F*_c_^2^)/3
2462 reflections	(Δ/σ)_max_ < 0.001
176 parameters	Δρ_max_ = 0.38 e Å^−^^3^
1 restraint	Δρ_min_ = −0.97 e Å^−^^3^

### Special details

**Table:**

Geometry. All e.s.d.'s (except the e.s.d. in the dihedral angle between two l.s. planes) are estimated using the full covariance matrix. The cell e.s.d.'s are taken into account individually in the estimation of e.s.d.'s in distances, angles and torsion angles; correlations between e.s.d.'s in cell parameters are only used when they are defined by crystal symmetry. An approximate (isotropic) treatment of cell e.s.d.'s is used for estimating e.s.d.'s involving l.s. planes.
Refinement. Refinement of *F*^2^ against ALL reflections. The weighted *R*-factor *wR* and goodness of fit *S* are based on *F*^2^, conventional *R*-factors *R* are based on *F*, with *F* set to zero for negative *F*^2^. The threshold expression of *F*^2^ > σ(*F*^2^) is used only for calculating *R*-factors(gt) *etc*. and is not relevant to the choice of reflections for refinement. *R*-factors based on *F*^2^ are statistically about twice as large as those based on *F*, and *R*- factors based on ALL data will be even larger.

### Fractional atomic coordinates and isotropic or equivalent isotropic displacement parameters (Å^2^)

**Table:**

	*x*	*y*	*z*	*U*_iso_*/*U*_eq_	
C1	0.8141 (4)	0.4519 (3)	0.0796 (4)	0.0606 (10)	
C2	0.7845 (5)	0.3933 (4)	−0.0224 (5)	0.0831 (15)	
H2	0.7044	0.4049	−0.0751	0.100*	
C3	0.8723 (6)	0.3186 (4)	−0.0457 (6)	0.0875 (15)	
H3	0.8519	0.2801	−0.1154	0.105*	
C4	0.9909 (5)	0.2983 (4)	0.0311 (5)	0.0800 (14)	
C5	1.0181 (6)	0.3562 (6)	0.1314 (6)	0.103 (2)	
H5	1.0970	0.3433	0.1851	0.124*	
C6	0.9322 (5)	0.4336 (5)	0.1561 (6)	0.0953 (18)	
H6	0.9544	0.4733	0.2245	0.114*	
C7	0.5844 (4)	0.4522 (3)	0.2993 (4)	0.0572 (9)	
C8	0.6858 (5)	0.4779 (3)	0.3929 (4)	0.0654 (11)	
H8	0.7506	0.5271	0.3806	0.079*	
C9	0.6891 (5)	0.4292 (3)	0.5048 (4)	0.0640 (10)	
C10	0.5946 (5)	0.3574 (4)	0.5250 (4)	0.0684 (11)	
C11	0.4920 (5)	0.3339 (4)	0.4311 (5)	0.0769 (13)	
H11	0.4256	0.2859	0.4438	0.092*	
C12	0.4885 (4)	0.3809 (3)	0.3209 (4)	0.0648 (11)	
H12	0.4195	0.3644	0.2589	0.078*	
C13	1.0877 (7)	0.2150 (5)	0.0040 (8)	0.118 (3)	
H13A	1.1115	0.2196	−0.0796	0.142*	
H13B	1.0414	0.1536	0.0158	0.142*	
H13C	1.1719	0.2187	0.0587	0.142*	
N1	0.5735 (4)	0.4999 (3)	0.1825 (4)	0.0644 (9)	
H1N	0.515 (4)	0.467 (3)	0.139 (4)	0.077*	
O1	0.7751 (4)	0.6143 (3)	0.1958 (3)	0.0814 (10)	
O2	0.6338 (3)	0.5836 (2)	−0.0009 (3)	0.0746 (9)	
Cl1	0.81491 (16)	0.46318 (12)	0.61972 (13)	0.0981 (6)	
Cl2	0.59783 (18)	0.29657 (12)	0.66355 (13)	0.1038 (6)	
S1	0.70098 (11)	0.54753 (8)	0.11309 (10)	0.0632 (4)	

### Atomic displacement parameters (Å^2^)

**Table:**

	*U*^11^	*U*^22^	*U*^33^	*U*^12^	*U*^13^	*U*^23^
C1	0.051 (2)	0.076 (3)	0.053 (2)	−0.0054 (18)	−0.0029 (16)	0.0103 (19)
C2	0.075 (3)	0.095 (3)	0.075 (3)	0.018 (3)	−0.019 (2)	−0.017 (3)
C3	0.090 (3)	0.088 (3)	0.084 (4)	0.013 (3)	0.000 (3)	−0.011 (3)
C4	0.069 (3)	0.083 (3)	0.090 (4)	0.009 (2)	0.018 (2)	0.023 (3)
C5	0.069 (3)	0.149 (6)	0.088 (4)	0.034 (3)	−0.016 (3)	−0.001 (4)
C6	0.063 (3)	0.141 (5)	0.077 (4)	0.017 (3)	−0.018 (2)	−0.019 (3)
C7	0.054 (2)	0.065 (2)	0.053 (2)	0.0078 (17)	0.0068 (16)	−0.0095 (18)
C8	0.062 (2)	0.071 (3)	0.062 (3)	−0.0084 (19)	−0.0018 (18)	−0.002 (2)
C9	0.064 (2)	0.070 (3)	0.057 (2)	0.004 (2)	0.0003 (18)	−0.008 (2)
C10	0.078 (3)	0.072 (3)	0.057 (2)	−0.003 (2)	0.018 (2)	−0.005 (2)
C11	0.072 (3)	0.086 (3)	0.076 (3)	−0.016 (2)	0.020 (2)	−0.012 (3)
C12	0.055 (2)	0.083 (3)	0.057 (2)	−0.004 (2)	0.0075 (17)	−0.015 (2)
C13	0.102 (4)	0.108 (5)	0.151 (7)	0.037 (4)	0.043 (4)	0.032 (4)
N1	0.0579 (19)	0.073 (2)	0.061 (2)	−0.0018 (16)	0.0008 (15)	0.0013 (18)
O1	0.094 (2)	0.078 (2)	0.072 (2)	−0.0218 (17)	0.0003 (17)	−0.0074 (17)
O2	0.0768 (19)	0.077 (2)	0.068 (2)	0.0037 (15)	−0.0080 (15)	0.0107 (16)
Cl1	0.0977 (10)	0.1188 (12)	0.0725 (9)	−0.0217 (8)	−0.0235 (7)	0.0056 (7)
Cl2	0.1393 (13)	0.1090 (11)	0.0655 (8)	−0.0192 (9)	0.0224 (8)	0.0143 (7)
S1	0.0652 (6)	0.0664 (7)	0.0567 (6)	−0.0025 (4)	−0.0028 (4)	0.0024 (5)

### Geometric parameters (Å, °)

**Table:**

C1—C6	1.366 (6)	C8—H8	0.9300
C1—C2	1.378 (7)	C9—C10	1.361 (6)
C1—S1	1.751 (5)	C9—Cl1	1.723 (4)
C2—C3	1.356 (7)	C10—C11	1.392 (7)
C2—H2	0.9300	C10—Cl2	1.720 (5)
C3—C4	1.377 (8)	C11—C12	1.359 (7)
C3—H3	0.9300	C11—H11	0.9300
C4—C5	1.355 (8)	C12—H12	0.9300
C4—C13	1.509 (7)	C13—H13A	0.9600
C5—C6	1.377 (8)	C13—H13B	0.9600
C5—H5	0.9300	C13—H13C	0.9600
C6—H6	0.9300	N1—S1	1.621 (4)
C7—C12	1.368 (6)	N1—H1N	0.83 (3)
C7—C8	1.390 (6)	O1—S1	1.426 (3)
C7—N1	1.425 (6)	O2—S1	1.435 (3)
C8—C9	1.385 (6)		
			
C6—C1—C2	119.3 (5)	C8—C9—Cl1	118.2 (4)
C6—C1—S1	120.0 (4)	C9—C10—C11	118.7 (4)
C2—C1—S1	120.7 (3)	C9—C10—Cl2	121.7 (4)
C3—C2—C1	119.9 (5)	C11—C10—Cl2	119.6 (4)
C3—C2—H2	120.1	C12—C11—C10	120.3 (4)
C1—C2—H2	120.1	C12—C11—H11	119.9
C2—C3—C4	121.7 (6)	C10—C11—H11	119.9
C2—C3—H3	119.1	C11—C12—C7	121.3 (4)
C4—C3—H3	119.1	C11—C12—H12	119.4
C5—C4—C3	117.7 (5)	C7—C12—H12	119.4
C5—C4—C13	121.1 (6)	C4—C13—H13A	109.5
C3—C4—C13	121.2 (6)	C4—C13—H13B	109.5
C4—C5—C6	121.8 (5)	H13A—C13—H13B	109.5
C4—C5—H5	119.1	C4—C13—H13C	109.5
C6—C5—H5	119.1	H13A—C13—H13C	109.5
C1—C6—C5	119.6 (5)	H13B—C13—H13C	109.5
C1—C6—H6	120.2	C7—N1—S1	126.8 (3)
C5—C6—H6	120.2	C7—N1—H1N	105 (4)
C12—C7—C8	119.1 (4)	S1—N1—H1N	116 (4)
C12—C7—N1	118.5 (4)	O1—S1—O2	119.3 (2)
C8—C7—N1	122.3 (4)	O1—S1—N1	108.2 (2)
C9—C8—C7	119.2 (4)	O2—S1—N1	104.04 (19)
C9—C8—H8	120.4	O1—S1—C1	109.0 (2)
C7—C8—H8	120.4	O2—S1—C1	108.3 (2)
C10—C9—C8	121.4 (4)	N1—S1—C1	107.4 (2)
C10—C9—Cl1	120.4 (4)		
			
C6—C1—C2—C3	−0.1 (9)	Cl1—C9—C10—Cl2	−1.0 (6)
S1—C1—C2—C3	−179.0 (5)	C9—C10—C11—C12	1.2 (7)
C1—C2—C3—C4	0.9 (10)	Cl2—C10—C11—C12	−179.7 (4)
C2—C3—C4—C5	−0.4 (9)	C10—C11—C12—C7	−0.2 (7)
C2—C3—C4—C13	180.0 (6)	C8—C7—C12—C11	−1.1 (6)
C3—C4—C5—C6	−0.8 (9)	N1—C7—C12—C11	−179.0 (4)
C13—C4—C5—C6	178.8 (6)	C12—C7—N1—S1	−151.4 (3)
C2—C1—C6—C5	−1.1 (9)	C8—C7—N1—S1	30.8 (6)
S1—C1—C6—C5	177.8 (5)	C7—N1—S1—O1	−53.2 (4)
C4—C5—C6—C1	1.6 (10)	C7—N1—S1—O2	178.9 (4)
C12—C7—C8—C9	1.4 (6)	C7—N1—S1—C1	64.3 (4)
N1—C7—C8—C9	179.2 (4)	C6—C1—S1—O1	19.7 (5)
C7—C8—C9—C10	−0.4 (7)	C2—C1—S1—O1	−161.4 (4)
C7—C8—C9—Cl1	−179.4 (3)	C6—C1—S1—O2	150.9 (4)
C8—C9—C10—C11	−0.8 (7)	C2—C1—S1—O2	−30.2 (5)
Cl1—C9—C10—C11	178.2 (4)	C6—C1—S1—N1	−97.4 (5)
C8—C9—C10—Cl2	−180.0 (4)	C2—C1—S1—N1	81.6 (4)

### Hydrogen-bond geometry (Å, °)

**Table:**

*D*—H···*A*	*D*—H	H···*A*	*D*···*A*	*D*—H···*A*
N1—H1N···O2^i^	0.83 (3)	2.10 (3)	2.908 (5)	166 (5)

Symmetry codes: (i) −*x*+1, −*y*+1, −*z*.
